# Protein Kinase A Regulates Platelet Phosphodiesterase 3A through an A-Kinase Anchoring Protein Dependent Manner

**DOI:** 10.3390/cells13131104

**Published:** 2024-06-26

**Authors:** Jawad S. Khalil, Robert Law, Zaher Raslan, Lih T. Cheah, Matthew S. Hindle, Ahmed A. Aburima, Mark T. Kearney, Khalid M. Naseem

**Affiliations:** 1 Leeds Institute of Cardiovascular & Metabolic Medicine, University of Leeds, Leeds LS2 9JT, UK; j.s.khalil@leeds.ac.uk (J.S.K.); zaherraslan@hotmail.com (Z.R.); l.t.cheah@leeds.ac.uk (L.T.C.); m.hindle@leedsbeckett.ac.uk (M.S.H.); m.t.kearney@leeds.ac.uk (M.T.K.); 2 Hull York Medical School, University of Hull, Hull HU6 7EL, UK; robdavelaw@gmail.com (R.L.); ahmed.aburima@hyms.ac.uk (A.A.A.)

**Keywords:** platelets, phosphodiesterase 3A, AKAP7

## Abstract

Platelet activation is critical for haemostasis, but if unregulated can lead to pathological thrombosis. Endogenous platelet inhibitory mechanisms are mediated by prostacyclin (PGI_2_)-stimulated cAMP signalling, which is regulated by phosphodiesterase 3A (PDE3A). However, spatiotemporal regulation of PDE3A activity in platelets is unknown. Here, we report that platelets possess multiple PDE3A isoforms with seemingly identical molecular weights (100 kDa). One isoform contained a unique N-terminal sequence that corresponded to PDE3A1 in nucleated cells but with negligible contribution to overall PDE3A activity. The predominant cytosolic PDE3A isoform did not possess the unique N-terminal sequence and accounted for >99% of basal PDE3A activity. PGI_2_ treatment induced a dose and time-dependent increase in PDE3A phosphorylation which was PKA-dependent and associated with an increase in phosphodiesterase enzymatic activity. The effects of PGI_2_ on PDE3A were modulated by A-kinase anchoring protein (AKAP) disruptor peptides, suggesting an AKAP-mediated PDE3A signalosome. We identified AKAP7, AKAP9, AKAP12, AKAP13, and moesin expressed in platelets but focussed on AKAP7 as a potential PDE3A binding partner. Using a combination of immunoprecipitation, proximity ligation techniques, and activity assays, we identified a novel PDE3A/PKA RII/AKAP7 signalosome in platelets that integrates propagation and termination of cAMP signalling through coupling of PKA and PDE3A.

## 1. Introduction

Blood platelets and their activation in response to vascular injury play a critical role in haemostasis, but if uncontrolled, it can develop into pathological thrombi. Platelet activation is regulated by endothelial-derived mediators, nitric oxide and prostacyclin (PGI_2_), through the activation of cyclic nucleotide signalling pathways [1]. PGI_2_ mediates its effects through a pathway that involves enzymes that generate, propagate, and terminate cAMP signalling in a coordinated fashion. We have shown that elevated cAMP and activation of its effector protein kinase A (PKA) reduces Ca^2+^ mobilisation, dense granule secretion, spreading, integrin αIIbβ3 activation and aggregation *in vitro*, and thrombosis *in vivo* [2,3,4,5,6], through the phosphorylation of a multitude of substrates [7]. Competent cAMP signalling creates an activatory threshold that ensures that platelets remain quiescent as they pass through the circulation and perturbation of the system potentially leads to platelet hyperactivity and thrombosis.

While it has been established for over 50 years that the cAMP signalling system is critical for the regulation of platelet function, our knowledge of how the systems that generate, propagate, and terminate are integrated is limited. Human platelets express two adenylyl cyclases (AC3 and AC6), four regulatory PKA subunits (RIα, RIβ, RIIα, RIIβ), three PKA catalytic subunits (Cα, Cβ and Cγ), and three phosphodiesterases (PDE2, PDE3A and PDE5) [8,9]. Recently, we have identified PDE3A as a potential signalling node that controls haemostasis, but under disease conditions, may drive maladaptive platelet hyperactivity and thrombosis [5,6]. PDE2 and PDE3A both show hydrolytic activity against cAMP, but pharmacological studies indicate that PDE3A plays the dominant role in controlling platelet cAMP signalling. Inhibition of PDE2 increases cAMP without affecting platelet function, while inhibition of PDE3A increased cAMP, PKA-dependent signalling, and inhibition of Ca^2+^ mobilisation [10,11]. The pharmacological inhibition or genetic deletion of PDE3A results in PGI_2_-hypersensitivity coupled to diminished platelet activation *in vitro* and thrombosis *in vivo*, highlighting its critical importance in controlling platelet activity [5]. Given the potential importance of PDE3A in platelet activity, it presents an attractive target for controlling hyperactivity in disease. Cilostazol, a PDE3A inhibitor, has been used clinically in the context of intermittent claudication where it can improve walking times. In experimental systems, Cilostazol inhibits platelet activation, aggregation, platelet–leukocytes and platelet–endothelium interactions through its ability to potentiate the effects of PGI_2_ [12,13,14]. However, its use as an antiplatelet agent has been limited because meta-analyses have shown side effects such as tachycardia, palpitation, or hypotension, with contraindications of heart failure, previous myocardial infarction, and unstable angina [15]. These side effects may reflect Cilostazol targeting the PDE3A catalytic site and therefore systematically inhibiting all PDE3A isoforms. Nevertheless, an agent that targets specific isoforms of PDE3A may be an effective way of controlling platelet activation. This more nuanced approach to targeting PDE isoforms requires an improved understanding of PDE3A biology in platelets.

Much of the data regarding the role of cAMP in platelet function has been gained from *in vitro* studies, using cAMP mimetics that act as global cAMP modulators or bypass AC, as well as pharmacological inhibitors that have off-target effects. Consequently, a precise understanding of the relationship between specific AC, PKA and PDE isoforms, individual signalling targets, and their compartmentalisation by AKAPs is still lacking. In many cells, cAMP modulates such distinct aspects of cell function through the selective coupling of its signalling complexes to specific substrates or regions within the cell, often through A-kinase anchoring proteins (AKAPs) [16,17,18,19]. The role of AKAPs in platelet biology is unclear although transcriptomic and proteomic studies suggest the presence of AKAP1, AKAP2, AKAP7, AKAP8, AKAP9, AKAP10, AKAP11, AKAP12 (Gravin), AKAP13, moesin, Ezrin, Rab32, and smAKAP in human platelets [9,20]. Previously, we have demonstrated a role for AKAPs in cAMP-mediated control of platelet activation [4], although their role in platelet PDE biology is unknown and requires clarification. To achieve this, we set out to examine some of the factors regulating platelet PDE3A activity and in particular to determine if PDE3A activity was regulated by a specific signalling complex as described for cardiomyocytes where PDE3A forms a complex with PKA RII, AKAP18, and SERCA2, to modulate heart contractility and sarcoplasmic reticulum Ca^2+^ content [21]. While earlier studies in platelets have identified PKA, PKB, and PKC mediated phosphorylation and activity [22,23], the identification of the PKA isoform responsible for the effects and how this is scaffolded to PDE3A remains elusive. Here, we describe a novel signalling complex in platelets composed of PDE3A, PKA RII, and AKAP7, the first such complex to be identified in platelets.

## 2. Materials and Methods

### 2.1. Reagents

Antibody suppliers were as follows: PKA RI, PKA RII, and PKAcat were from BD Biosciences. β3 integrin was from Santa Cruz (Insight Biotech Ltd., Wembley, UK). SLP-76, GAPDH, and phosphoVASP ser^157^ were from Cell Signalling Technology (Leiden, The Netherlands). PDE3A(CT), PDE3A(UNT), PDE3A(NT), and phosphoPDE3A antibodies were from the MRC Unit (Dundee University, Dundee, Scotland). AKAP7 antibody was supplied by Prof. Enno Klussmann (Max Delbrück Centre for Molecular Medicine, Berlin, Germany). DC protein assay kit II, polyvinylidene difluoride (PVDF), and bis-acrylamide solution were obtained from Bio-Rad Laboratories Ltd. (Hertfordshire, UK). Phosphodiesterase activity assay was obtained from Enzo life sciences (Exeter, UK). Amersham cAMP biotrack enzyme-immunoassay (EIA) system, fetal bovine serum (FBS), and L-glutamine were obtained from GE Healthcare Life Sciences (Buckinghamshire, UK). Crosslink co-immunoprecipitation kit and RPMI 1640 medium were obtained from ThermoFisher scientific (Loughborough, UK). 8-(4-Chlorophenylthio) adenosine-3′,5′-cyclic monophosphate (8-CPT-cAMP) and 8-CPT-cAMP Rp isomer (RP-8-CPT-cAMP) were obtained from Biology Life Science (Bremen, Germany). Collagen reagent Horm was from Nycomed (Munich, Germany). Prostacyclin (PGI_2_) was from Cayman Chemical Company, (Cambridge, UK). AKAP HT31 inhibitor peptide (HT31) and its control peptide (HT31-P) were obtained from Promega (Southampton, UK). Forskolin, KT5720, phosphatase inhibitor cocktail, and protease inhibitor cocktail were obtained from Sigma-Aldrich Company Ltd. (Poole, UK). Milrinone was from Tocris Bioscience, (Bristol, UK). RI anchoring disruptor peptide (RIAD) and its scrambled version (RIAD scr) were supplied by Professor Kjetil Tasken (Centre for Molecular Medicine, Norway). 8-AHA-cAMP and EtOH-NH-Agarose beads were from BIOLOG Life Science Institute (Bremen, Germany). All other general reagents and kits were purchased from Sigma-Aldrich Company Ltd., Dorset, UK.

### 2.2. Platelet and Megakaryocyte Isolation

Blood was taken from drug-free volunteers by clean venepuncture using acid citrate dextrose (ACD; 29.9 mM sodium citrate, 113.8 mM glucose, 72.6 mM NaCl, and 2.9 mM citric acid, pH 6.4) as anticoagulant. Washed human platelets were prepared as described previously [3].

Murine blood was obtained from the inferior vena cava and was drawn into 1 mL syringes with 200 µL of ACD. Whole blood was diluted to a final volume of 2 mL with Modified Tyrodes buffer (0.5 mM MgCl_2_, 0.55 mM NaH_2_PO_4_, 2.7 mM KCl, 5 mM HEPES, 5.6 mM glucose, 7 mM NaHCO_3_, and 150 mM NaCl, pH 7.4). Diluted whole blood was centrifuged at 100× *g* for 5 min to isolate platelet-rich plasma (PRP); blood was diluted further to a final volume of 1 mL and centrifuged again at 100× *g* for 5 min. The final PRP was removed and pooled, then centrifuged at 1000× *g* for 6 min. The platelet pellet was then resuspended in Modified Tyrodes buffer and adjusted to 5 × 10^8^ platelets/mL.

Murine megakaryocyte lysates were a kind gift from Dr Simon Calaminus (University of Hull, UK). Megakaryocytes were isolated from the bone marrow of >6 weeks old mice from their hind legs. Both tibiae and femurs were flushed followed by depletion of immune cells with immunomagnetic beads. The cell-depleted population was then cultured in serum-free medium supplemented with 2 mM L-glutamine, penicillin/streptomycin, and 20 ng/mL murine stem cell factor at 37 °C. After 7 days of differentiation, mature megakaryocytes were purified with a 1.5%/3% bovine serum albumin gradient for 45 min at room temperature and constituted >60% of the enriched cell population as reported previously [24].

### 2.3. Platelet Aggregation

Washed human platelets (2.5 × 10^8^ platelets/mL) were incubated with milrinone (10 µM) at 37 °C for 20 min followed by 1 min treatment with PGI_2_ (10 nM) before stimulated with collagen (10 µg/mL) and monitored under constant stirring for 4 min using a Chronolog Dual Channel Platelet Aggregometer.

### 2.4. Measurement of cAMP

Washed platelets (2 × 10^8^ platelets/mL) were treated with PGI_2_ (10 nM and 100 nM) for 1 min, in the presence and absence of inhibitors, before termination with lysis buffer. cAMP levels were assayed with a commercial enzyme immunoassay system and expressed as fmol cAMP/1 × 10^7^ platelets.

### 2.5. PKA Activity Assay

PKA activity associated with AKAP7 immunoprecipitates was measured by phosphorylation (increase negative charge) of the synthetic PKA substrate kemptide as described previously [4].

### 2.6. Measurement of PDE Activity

To measure intracellular PDE activity, we used a commercially available non-radioactive colorimetric assay. Washed platelets (5 × 10^8^ platelet/mL) were treated as appropriate, lysed in 2× PDE extraction buffer (150 mM sodium chloride, 50 mM HEPES, 20% glycerol (*v*/*v*), 10% Igepal (*v*/*v*), 1 mM, EDTA, 1:200 phosphatase inhibitor cocktail (*v*/*v*), and protease inhibitor cocktail 1:100 (*v*/*v*)), and immediately placed on ice. PDE3A was immunoprecipitated from 500 μg of lysate protein using 1μg of PDE3A antibody or IgG. Immunoprecipitates were incubated with 5′-nucleotidase and cAMP (0.5 mM) substrate at 37 °C for 1 hr and the production of 5′-AMP was measured following the manufacturer’s instructions. Activity was expressed as fmol AMP/min/1 × 10^7^ platelets or relative luminescence units (RLUs) and in some cases normalized to control value to account for variations in basal activity between individual platelet donors.

### 2.7. Subcellular Fractionation

For subcellular fractionation, washed platelets (5 × 10^8^ platelets/mL) were lysed by addition of equal amounts of 2× fractionation buffer (320 mM sucrose, 4 mM HEPES, 0.5 mM Sodium orthovanadate) supplemented with protease and phosphatase inhibitors, followed by 5 freeze–thaw cycles. Lysates were subjected to ultracentrifugation at 100,000× *g* for 90 min at 4 °C. Supernatants (cytosolic fraction) were aspirated, and pellets (plasma and intracellular membranes) were resuspended with IP lysis buffer [3]. Protein concentrations were measured, and equal amounts of protein were analysed. Fractionated platelet lysates were subjected to SDS-PAGE and immunoblotting. β3, GAPDH, and SLP-76 were used to validate the fractionation.

### 2.8. Immunoprecipitation, cAMP Pull Down, and Immunoblotting

Washed platelets (3–5 × 10^8^ platelets/mL) were incubated with apyrase (2 U/mL), indomethacin (10 µM), and EGTA (1 mM) before treatment with PGI_2_, forskolin or 8-CPT-6-Phe-cAMP. Platelets were lysed with Laemmli buffer and proteins were separated by SDS-PAGE before transfer to PVDF membranes. In some cases, platelets were incubated with PKA inhibitors or AKAP disruptor peptides before addition of PGI_2_. For immunoprecipitation, washed platelets (8 × 10^8^ platelets/mL) were lysed with ice-cold lysis buffer and proteins were immunoprecipitated using standard protocols [3]. Proteins were separated by SDS-PAGE and transferred to PVDF membranes. Membranes were blocked for 60 min with 5% milk in Tris-buffered-saline-Tween (0.1%) (TBS-T), then incubated with the indicated antibody. Immunoblots were processed as described previously [3]. For cAMP pull down experiments, platelet lysates were incubated with agarose-beads linked to 8-AHA-cAMP or EtOH-NH. Beads were incubated with lysates overnight at 4 °C with mixing. Beads were then washed, and proteins eluted by boiling in Laemmli buffer for 5 min and analysed by SDS-PAGE followed by immunoblotting.

### 2.9. Mass Spectrometry

Washed platelets (8 × 10^8^ platelets/mL) were lysed with ice-cold IP lysis buffer and PDE3A proteins were immunoprecipitated with PDE3A (CT) and PDE3A (UNT) antibodies as described above. Samples were subjected to SDS-PAGE prior to Coomassie staining of gels. Bands at 100 kDa and around 130 kDa were excised alongside corresponding bands in control IgG lane and analysed via mass spectrometry.

### 2.10. Immunofluorescence and Duolink^TM^ Proximity Ligation Assay

The cellular distribution of PKA isoforms and their interactions with other proteins were visualised by standard immunofluorescence or in combination with a DuolinkTM proximity ligation assay (PLA) [25] as described previously. Four fields of view were analysed for each condition. In brief, each field of view underwent automatic thresholding and particles were analysed for count and distribution (FIJI, ImageJ 1.52c).

### 2.11. Statistical Analysis

Data were analysed by Graphpad Prism 8 (La Jolla, CA, USA) and are presented as means ± standard error of the mean (SEM) of at least 3 different experiments. Differences between groups were calculated using the Student’s *t*-test or ANOVA and statistical significance was taken at *p* ≤ 0.05.

## 3. Results

### 3.1. Identification of PDE3A Isoforms in Platelets

**Figure 1:**
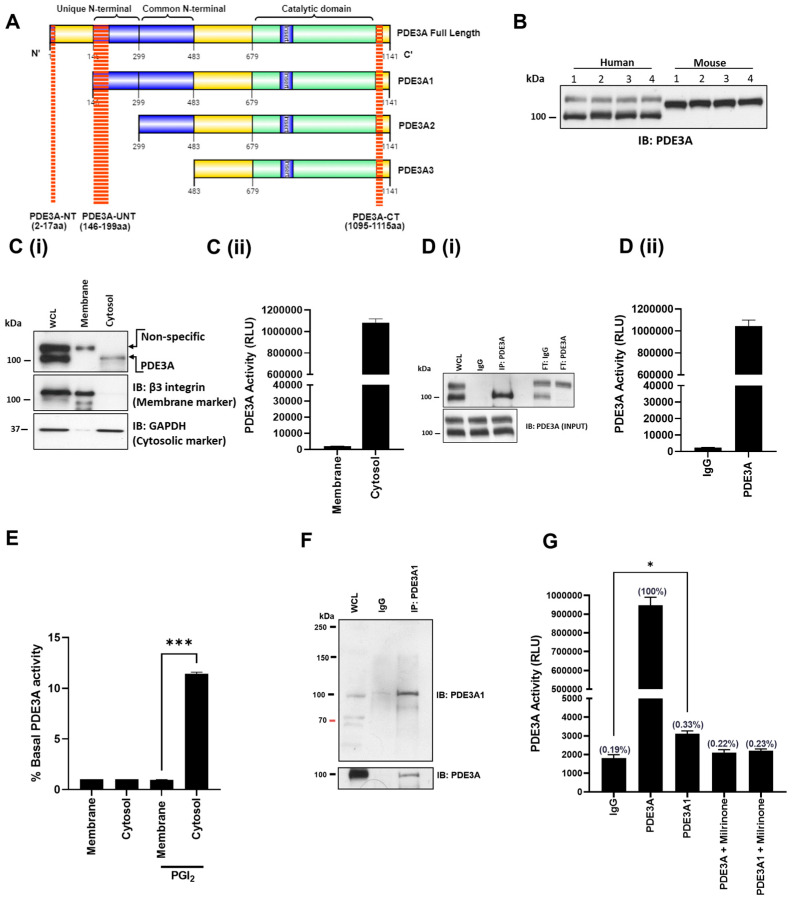
Identification of PDE3A isoforms in blood platelets. (**A**) Schematic representation of PDE3A isoforms with N- and C-terminal domain architecture and antibody binding sites for PDE3A(NT), PDE3A(UNT), and PDE3A(CT). (**B**) Platelet lysates (5 × 10^8^ platelets/mL) from four human donors and four mice (C57BL/6 to8 weeks) were separated by SDS-PAGE and immunoblotted for total PDE3A using PDE3A(CT) antibody. (**C(i)**) Washed human platelets (5 × 10^8^ platelets/mL) were fractionated using liquid nitrogen and analysed for PDE3A localisation using immunoblotting. Blots were stripped and reprobed for the membrane marker, β3, and cytosolic marker, GAPDH, as fraction controls. Representative immunoblots of 3 independent experiments. (**C(ii)**) Fractionated PDE3A (500 µg protein) was immunoprecipitated and subjected to PDE3A activity assay. (**D(i**)) PDE3A was immunoprecipitated from washed human platelet lysates (500 μg protein) with PDE3A(CT) antibody (2 mg) or IgG and immunoblotted and assayed for PDE3A activity, in presence and absence of milrinone (10 μM, 20 min). (**D(ii)**) Data are expressed as relative luminescence units (RLU) and representative of 4 independent experiments. (**E**) Washed human platelets (5 × 10^8^ platelets/mL) were treated with PGI_2_ (100 nM) before fractionation using liquid nitrogen, immunoprecipitation of PDE3A was assayed for PDE activity. Data are presented as percentage increase over basal activity for each fraction (*n* = 3; *** *p* < 0.001). (**F**) Human platelet lysates (500 µg protein) were incubated with PDE3A(UNT) antibody (10 μg) for 1 hr before incubated overnight with 25 µL protein G Sepharose beads slurry. Following incubation, immunoprecipitated proteins were isolated and immunoblotted with PDE3A(UNT) and PDE3A(CT) antibodies. Whole cell lysates were run as input controls. Representative immunoblots of 3 independent experiments. (**G**) Respective immunoprecipitated PDE3A proteins from washed human platelet lysates (500 μg protein) and assayed for PDE3A activity, in presence and absence of milrinone (10 μM, 20 min). Data are expressed as relative luminescence units (RLU) and representative of 3 independent experiments (* *p* < 0.05 compared to IgG).

PDE3A plays a critical role in cAMP signalling and cAMP-mediated inhibition of platelet function. Inhibition of platelet PDE3A activity with milrinone (10 µM) led to an increase in platelet cAMP, PKA signalling, and inhibition of platelet aggregation, but also potentiated the inhibitory effects of PGI_2_ (Figure S1). However, it is unclear if the effects of milrinone occur through one or more PDE3A isoforms. Figure 1A shows the amino acid sequences for different PDE3A isoforms in cardiomyocytes [26]. To investigate the presence of different PDE3A isoforms in platelets, three antibodies have been generated. First, a PDE3A(NT) antibody raised against the beginning of N-terminus (amino acids 2–17; MRC phosphorylation Unit, Dundee, UK) was used to determine if a full length PDE3A is present. However, no band was observed, indicating that platelets may not express full length PDE3A, or the binding site was somehow not accessible by the antibody. A second PDE3A antibody was raised against amino acids 1095–1110 at C-Terminus (PDE3A(CT); MRC phosphorylation Unit, Dundee, UK), which would label all PDE3A isoforms. Immunoblotting of human and murine platelet lysates with this antibody suggested the presence of different PDE3A isoforms (Figure 1B). In human platelets, we observed bands at 100 kDa and 130 kDa, while in murine lysates, we only observed one band at approximately 120 kDa. These data suggested the possibility of distinct PDE3A isoforms in human platelets. To explore this, we performed subcellular fractionation and observed that the 100 kDa band was localised in the cytosol, while the 130 kDa band was present in the membrane fraction (Figure 1Ci). Interestingly, we only detected PDE3A activity in the cytosolic fraction (Figure 1Cii), suggesting that the 130 kDa may not be PDE3A. Immunoprecipitation experiments from human platelets using this PDE3A(CT) antibody found that only a single 100 kDa band was precipitated and this was associated with PDE3A activity; the 130 kDa band was found only in the flow-through (Figure 1Di,Dii). Similar data were observed using commercial PDE3A antibodies (NOVUS and Santa Cruz). To determine if the location of PDE3A activity was affected by increased cAMP, platelets were stimulated with PGI_2_ (100 nM), fractionated, and the activity measured. While PGI_2_ did cause a significant increase in PDE3A, this was confined to the cytosolic fraction (Figure 1E). To confirm these data, we performed the mass spectrometric analysis on these bands and confirmed that the 130 kDa band was not PDE3A (Figure S2 and Table S1).

To further explore the possibility that platelets may possess multiple isoforms of PDE3A, we generated another antibody raised against the Unique N-Terminus (amino acids 145–199) of PDE3A(UNT), which has been shown to be present in PDE3A1 but not in other isoforms (Figure 1A) [26]. Immunoprecipitation with this antibody followed by immunoblotting revealed a faint band for PDE3A at approximately 100 kDa after prolonged exposure, suggesting only minor levels of expression (Figure 1F). Mass spectrometry analysis of this band confirmed the presence of PDE3A (Figure S2 and Table S1). Immunoprecipitation of PDE3A with PDE3A(UNT) antibody followed by activity assays demonstrated a small but significant increase in activity (* *p* < 0.05), which was blocked by the general PDE3A inhibitor milrinone (10 µM) (Figure 1G). It is noteworthy that this level of activity using PDE3A(UNT) antibody was significantly less (approximately 0.33 ± 0.03%) than the overall PDE3A activity using the PDE3A(CT) antibody.

### 3.2. PDE3A Is Transiently Phosphorylated and Activated by PKA in Response to PGI_2_

**Figure 2:**
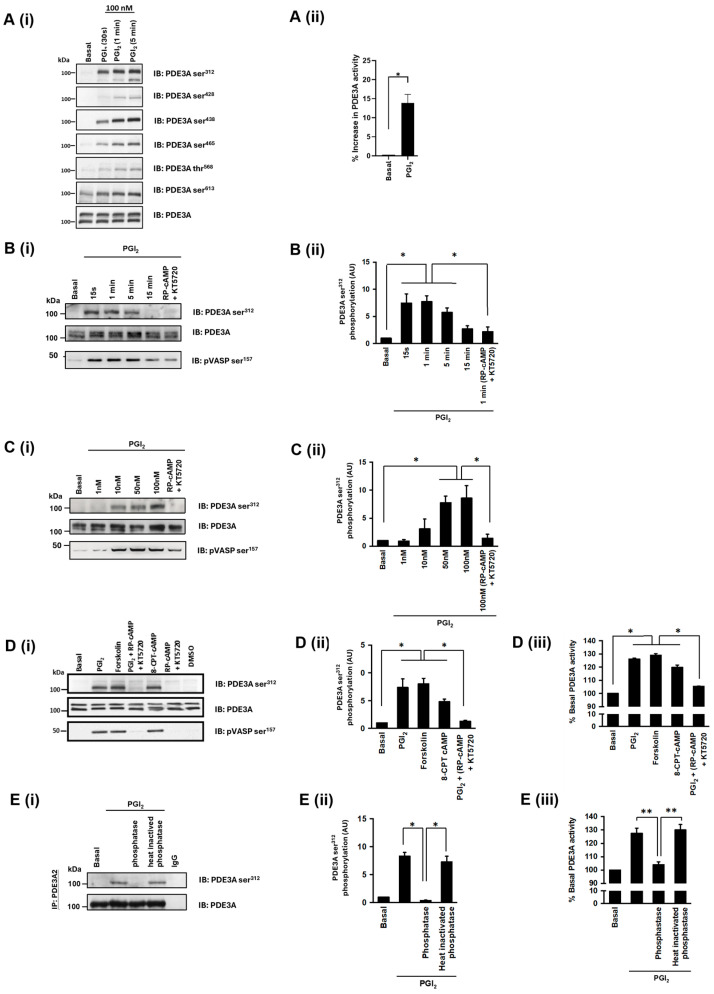
PGI_2_ mediated inhibition of platelets results in PDE3A phosphorylation. (**A(i)**) Washed human platelets (5 × 10^8^ platelets/mL) were stimulated with PGI_2_ (100 nM) at indicated time points before lysates were resolved by SDS-PAGE and immunoblotted for PDE3A phosphorylation at sites ser^312^, ser^428^, ser^438^, ser^465^, thr^568^ and and ser^613^. Blots were stripped and reprobed for PDE3A as loading control. Representative immunoblots of 3 independent experiments. (**A(ii)**) Platelets were treated with PGI_2_ (100 nM, 5 min) and PDE3A was immunoprecipitated from platelet lysates (500 μg protein) before measuring PDE3A activity. Data are presented as percentage increase in basal PDE3A activity (*n* = 3; * *p* < 0.05). (**B(i)**) As in (**A(i)**), except human platelets were stimulated with PGI_2_ (100 nM) for up to 15 min before resolved by SDS-PAGE and immunoblotted for phosphoPDE3A ser^312^, phosphoVASP ser^157^, and total PDE3A. In some cases, platelets were preincubated with PKA inhibitors RP-cAMP (500 μM) and KT5720 (10 μM) for 20 min prior to PGI_2_ treatment. Representative blots from 4 independent experiments. (**B(ii)**) PDE3A phosphorylation densitometric analysis expressed as arbitrary unit (AU). (**C(i)**) As in (**B**), except human platelets were stimulated with 1–100 nM PGI_2_ (1 min). Representative blots from 3 independent experiments. (**C(ii)**) PDE3A phosphorylation densitometric analysis. (**D(i)**) Washed human platelets (5 × 10^8^ platelets/mL) were treated with either PGI_2_ (100 nM, 2 min), Forskolin (10 μM, 5 min) or 8-CPT-cAMP (100 μM, 5 min). Human platelets were also incubated with PKA inhibitors RP-cAMP (500 μM) and KT5720 (10 μM) for 20 min prior to PGI_2_ (100 nM, 2 min) treatment. Human platelet lysates were resolved by SDS-PAGE and immunoblotted for phosphoPDE3A ser^312^, phosphoVASP ser^157^, and total PDE3A. Representative immunoblots of 3 independent experiments. (**D(ii)**) Densitometric analysis of phosphoPDE3A ser^312^. (**D(iii)**) As in (**D(ii)**), except PDE3A was immunoprecipitated from washed human platelet lysates (500 μg protein) and assayed for PDE3A activity (* *p* < 0.05). (**E**) Washed human platelets (5 × 10^8^ platelets/mL) were treated with PGI_2_ (100 nM, 2 min) before lysis and immunoprecipitation of PDE3A. Immunoprecipitates were incubated with active or heat-inactivated calf intestinal phosphatase for 1 hr at 37 °C. (**E(i)**) PDE3A ser^312^ phosphorylation was assessed by immunoblotting. Blots were stripped and reprobed for PDE3A as protein loading control. Representative immunoblots of 3 independent experiments. (**E(ii)**) Densitometric analysis of phosphoPDE3A ser^312^. (**E(iii)**) As in (**E(ii)**), except PDE3A immunoprecipitates were assayed for PDE3A activity (** *p* < 0.01).

Previously, it has been demonstrated that PDE3A is one of the numerous proteins that is phosphorylated by PKA in response to PGI_2_ [23]. We have shown the first evidence of AKAP regulation of PKA phosphorylation in platelets [4] and hypothesised that phosphorylation and activation of PDE3A may also be AKAP mediated. In the first instance, we sought to characterise this phosphorylation event. Consistent with previous studies, stimulation of platelets with PGI_2_ (100 nM) caused a time dependent increase in phosphorylation of PDE3A on multiple sites including phosphoPDE3A ser^312^, ser^428^, ser^438^, ser^465^, thr^568^, and ser^613^ (Figure 2Ai and Figure S3), but we were unable to confirm phosphorylation on other reported PDE3A phosphorylation sites including ser^293^, ser^294^, and. Similarly, immunoprecipitation of PDE3A followed by activity assays showed that PGI_2_ (100 nM, 5 min) significantly increased PDE3A activity compared to basal (Figure 2Aii).

We used the phosphorylation at ser^312^ (phosphoPDE3A ser^312^) to confirm the key role of cAMP signalling in the regulation of PDE3A. PGI_2_ (100 nM) induced a rapid and reversible phosphorylation of the PDE3A, with phosphorylation commencing in 15 s and returning to basal after 15 min (Figure 2Bi,Bii). The actions of PGI_2_ were also concentration dependent with significant phosphorylation at 50 nM and maximal at 100 nM (Figure 2Ci,Cii). These PGI_2_-mediated PDE3A phosphorylation events were significantly reduced by PKA inhibitors RP-cAMP (500 μM) and KT5720 (10 μM), respectively. As a positive control, PGI_2_ treatment induced phosphorylation of cytoskeleton-associated vasodilator-stimulated phosphoprotein (VASP), which is a marker of platelet inhibition. To establish the signalling mechanisms linking PGI_2_ to the phosphorylation of PDE3A, we used a number of pharmacological agents to modulate the cAMP-signalling pathway (Figure 2D). Pre-treatment of platelets with the PKA inhibitors RP-cAMP (500 μM) and KT5720 (10 μM) prior to PGI_2_ stimulation significantly reduced the phosphorylation of PDE3A ser^312^ (7-fold reduction) (Figure 2Di,Dii) and activity (22 ± 3% versus 6 ± 2%; *p* < 0.05, Figure 2Diii). The direct activator of AC, forskolin (10 µM), also induced a 7-fold increase in PDE3A ser^312^ phosphorylation and activity (29 ± 3% over basal, Figure 2Diii), confirming a central role of adenylyl cyclase. Moreover, the phosphorylation and activation of PDE3A was induced by the cell permeable activator of PKA, 8-CPT-cAMP (100 μM) (18 ± 4% over basal, Figure 2Diii). To confirm that phosphorylation was important for enzyme activity, we used calf intestinal phosphatase (CIP) to dephosphorylate the protein. Treatment of PDE3A with CIP reversed both the increase in enzyme activity and phosphorylation induced by PGI_2_ (Figure 2E). In contrast, heat-inactivated CIP had no effect. We were unable to detect phosphorylation of PDE3A1, likely because of the low overall yield. Therefore, consistent with previously published data, our data suggest that PKA phosphorylation of PDE3A increases the enzymatic activity in response to stimulation of cAMP signalling.

### 3.3. AKAP Disruption Reduces cAMP-Signalling-Mediated Activation of PDE3A

**Figure 3:**
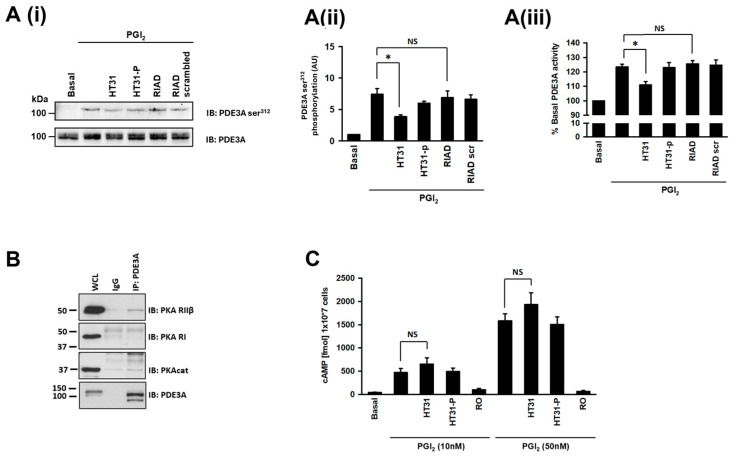
Effect of AKAP disruption on PDE3A activity and phosphorylation. (**A**) Washed human platelets (5 × 10^8^ platelets/mL) were incubated with either non-specific AKAP disruptor peptide HT31 (2 μM), the HT31 control peptide HT31-P (2 μM), PKA RI specific disruptor peptide, RIAD (2.5 μM), and RIAD control peptide RIAD-scrambled (2.5 μM) for 60 min prior to stimulation with PGI_2_ (100 nM, 2 min). Samples were immunoblotted for PDE3A ser^312^ phosphorylation and PDE3A as a loading control and analysed for PDE3A activity. (**A(i)**) Representative blots of 3 independent experiments. (**A(ii)**) PDE3A ser^312^ phosphorylation densitometric analysis of 3 independent experiments expressed as arbitrary units (AU) * *p* < 0.05. NS, not significant. (**A(iii)**) As in (**A(ii)**), except PDE3A immunoprecipitates were assayed for PDE3A activity (* *p* < 0.05). (**B**) Human platelet lysates (500 mg proteins) were incubated with PDE3A(CT) antibody (2 μg) to immunoprecipitate PDE3A. The PDE3A immunoprecipitates were then blotted for PKA RI and PKA RII on separate membranes. Immunoblots were stripped and reprobed for PDE3A as a loading control and PKA catalytic subunit (PKAcat) as a positive control. Representative blots from 3 independent experiments. (**C**) As in A, except human platelets were either pre-treated with 10 nM or 50 nM PGI_2_, lysed, and cAMP measured. In some cases, human platelets were preincubated with the IP receptor antagonist (RO1138452; 100 µM) prior to PGI_2_. Data are expressed as cAMP (fmol/10^7^ platelets) (*n* = 4).

We have previously demonstrated the first AKAP-dependent targeting of PKA in platelets [4] and therefore examined whether AKAPs also facilitated PKA targeting of PDE3A using cell permeable AKAP-uncoupling peptides. Preincubation of platelets with HT31 (2 μM), which uncouples both PKA RI and PKA RII from their associated AKAPs [27,28], significantly reduced PGI_2_-induced phosphorylation (Figure 3Ai,Aii), PGI_2_-mediated inhibition in aggregation (Figure S4), and activation of PDE3A (23 ± 5% versus 11 ± 4%; *p* < 0.05; Figure 3Aiii), while the control peptide HT31-P had no effect on either phosphorylation, aggregation, or activity. In contrast to HT31, the type-1 AKAP specific uncoupling peptide RIAD (RI anchoring disrupter) [29] or its scrambled control peptide did not affect the phosphorylation or enzyme activity. Having observed that disruption of PKA RII anchoring blunts PDE3A phosphorylation and activity, we reasoned that PKA RII should be associated with PDE3A. After immunoprecipitation of PDE3A, we immunoblotted for PKA RI and PKA RII, and consistent with the data generated using HT31, we found that RII but not RI was associated with PDE3A at least at basal level (Figure 3B). Given that PDE3A activity controls the availability of cAMP, we hypothesised that the uncoupling of PKA from PDE3A may influence overall cAMP concentrations. Incubation of platelets with HT31, but not HT31-P, caused an insignificant but small and consistent increase in platelet cAMP both under basal conditions and in response to PGI_2_ (Figure 3C). These data suggest that regulation of PDE3A activity may be facilitated by a type-2 specific AKAP and that phosphorylation of PDE3A was potentially performed by PKA RII. Furthermore, the uncoupling of PKA from an AKAP may modulate overall platelet cAMP concentrations.

### 3.4. AKAP7δ Is Expressed and Acts as an AKAP in Human Platelets

**Figure 4:**
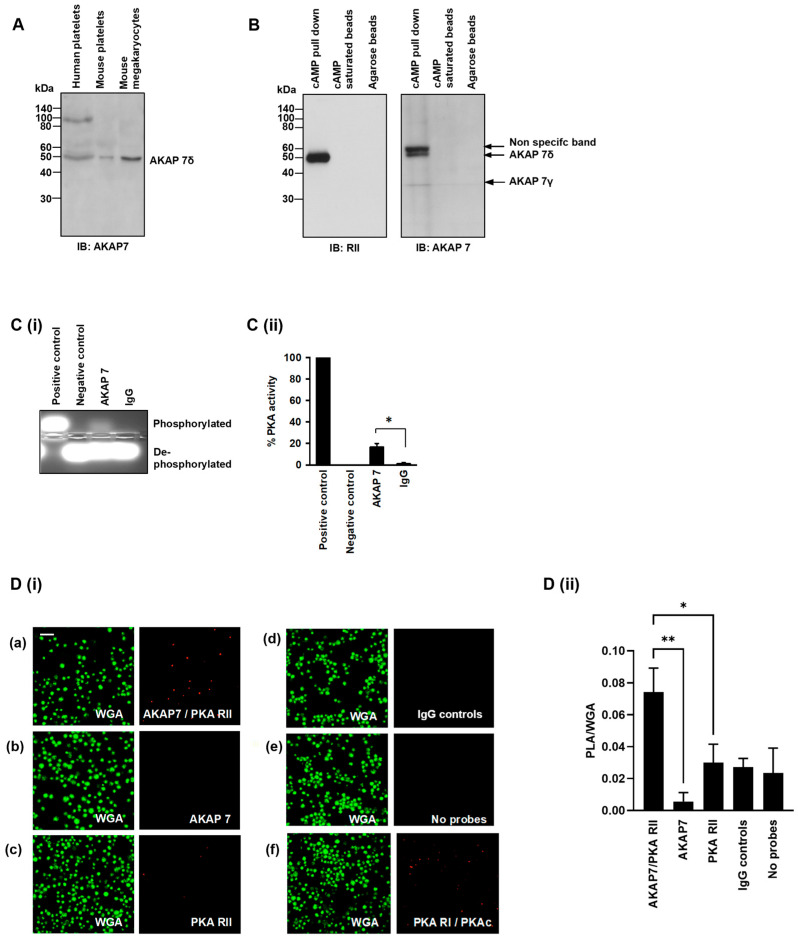
AKAP7 is expressed and acts as an AKAP in human platelets. (**A**) Washed human platelet, mouse platelet, and mouse megakaryocytes lysates (20 µg) were immunoblotted for AKAP7 expression. Representative of 3 independent experiments. (**B**) Washed platelet lysates (1 mg protein) were incubated with cAMP-bound agarose beads ± cAMP (25 µL). Following incubation, the resin, precipitated antigen, and co-immunoprecipitated proteins were isolated by centrifugation and immunoblotted for PKA RII or AKAP7 binding. Representative immunoblots from 3 independent experiments. (**C**) Washed platelet lysates (500 μg) were incubated with AKAP7 antibody (2 μg) coupled to protein G beads. AKAP7 immunoprecipitates were isolated using centrifugation and analysed for the ability to phosphorylate the synthetic PKA substrate kemptide as a measure of associated PKA. (**C(i)**) Representative agarose gel image and (**C(ii)**) Quantification of PKA activity expressed as percentage of PKA activity compared with IgG. Data representative of 3 independent experiments * *p* < 0.05. (**D**) Washed platelets (1 × 10^7^ platelets/mL) were subjected to proximity ligation assay to determine physical proximity of the key proteins. (**D(i)**) Platelets were co-stained with (**a**) PKA RII and AKAP7 antibodies, (**b**) AKAP7 alone, (**c**) PKA RII alone, (**d**) matched IgG controls, (**e**) no probes added, and (**f**) PKA RI and PKAc antibodies as a positive control. Platelet membranes were visualised using wheat germ agglutinin conjugated to Alexa Fluor 488 using fluorescence microscopy. Representative of 4 independent experiments. Scale bar: 10 µm. (**D(ii)**) Quantification of PLA of 4 independent experiments * *p* < 0.05, ** *p* < 0.01.

Analysis of recently published proteomic studies indicates that multiple AKAPs are expressed in platelets including AKAP1, AKAP2, AKAP7, AKAP8, AKAP9, AKAP10, AKAP11, AKAP12 (Gravin), AKAP13, moesin, Ezrin, Rab32, and smAKAP [20]. We have previously identified moesin [4], and our preliminary studies suggest the presence of AKAP7, AKAP9, AKAP12 and AKAP13 in human platelets (Figure 4 and Figure S5). Of these proteins, we were particularly attracted to AKAP7 (also known as AKAP18), since it has previously been shown to interact with PDE3A in cardiomyocytes [21,30]. Immunoblot analysis of human and murine platelet and mouse megakaryocyte whole cell lysates confirmed the presence of AKAP7δ (Figure 4A). Having confirmed the presence of AKAP7, we wished to determine that it had the capacity to interact with PKA. Our strategy was to employ the widely used cAMP affinity resins, an approach that allows the enrichment of PKA through the binding of the R-subunits to immobilised cAMP and the precipitation of associated proteins [31]. Under the conditions tested, the cAMP linked beads precipitated PKA RII (Figure 4B, left panel) but, critically, also pulled down AKAP7δ and a small amount of AKAP7γ (Figure 4B, right panel), confirming that AKAP7 acts as a PKA-binding protein in human platelets. A non-specific band of 60 kDa was also observed, as has been noted previously [32]. We then reasoned that if AKAP7 was associated with PKA, then it should possess a PKA activity (24). AKAP7 was immunoprecipitated from platelet lysates and the ability to phosphorylate kemptide, a synthetic PKA substrate, was examined [33]. Immunoprecipitated AKAP7 possessed a 17 ± 3%-fold higher PKA activity than with immunoprecipitated IgG control (Figure 4Cii), confirming an associated PKA activity. To determine whether the association between AKAP7 and PKA RII occurred in intact platelets, we performed proximity ligation assays. This approach showed that AKAP7 associates with PKA RII in platelets (Figure 4D). Association between PKA RI and PKAcat serves as a positive control (Figure 4Di(f)).

### 3.5. PDE3A Exists in a Complex with PKA Type II and AKAP7δ in Platelets

**Figure 5:**
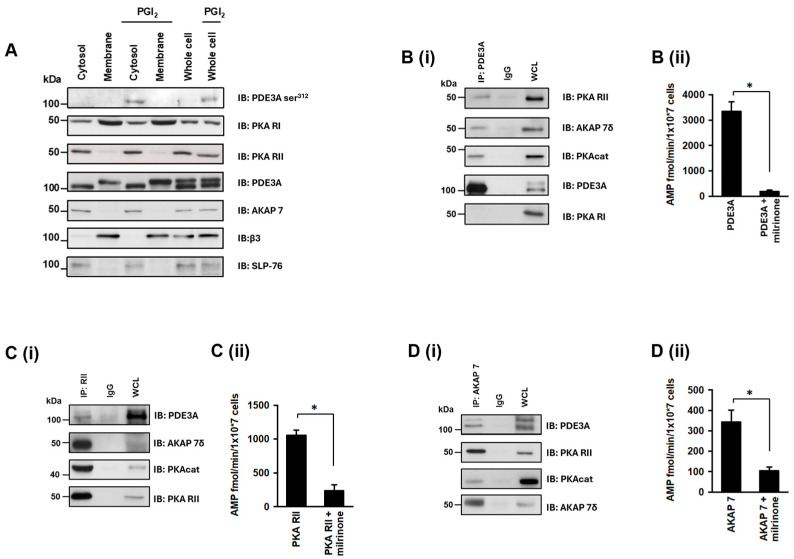
Identification of a PKA RII/AKAP7/PDE3A signalling complex in human platelets. (**A**) Washed human platelets (7 × 10^8^ platelets/mL) were stimulated with PGI_2_ (100 nM, 2 min), lysed, and centrifuged into membrane and cytosolic fractions. Fractions were immunoblotted for phosphoPDE3A ser^312^, PKA RI, PKA RII, AKAP7, β3, and SLP-76. (**B**) Washed human platelet lysates (1 mg proteins) were incubated with PDE3A antibody (5 μg) covalently coupled to an amine-reactive resin. PDE3A immunoprecipitates were immunoblotted for PKA RII and PKA RI and then reprobed for PDE3A as a loading control. Similarly, human platelet lysates (1 mg proteins) were incubated with (**C**) PKA RII antibody (5 μg), and (**D**) AKAP7 antibody (5 μg) covalently coupled to amine-reactive resin. Following incubation, co-immunoprecipitated proteins were isolated and immunoblotted for (**C(i)**) PDE3A, AKAP7, PKAcat, and PKA RII; and (**D(i)**) PDE3A, PKA RII, PKAcat, and AKAP7. Representative immunoblots of 3 independent experiments. (**B(ii)**,**C(ii)**,**D(ii)**) Respective co-immunoprecipitated proteins (500 μg protein) were assayed for PDE3A activity, in the presence and absence of milrinone (10 μM, 20 min). Data representative of 3 independent experiments * *p* < 0.01.

We next sought to establish the existence of the molecular complex that regulated PDE3A activity in platelets and whether this involved AKAP7. In the first instance, we fractionated platelets to determine the location of components of the complex. Under basal conditions, PDE3A, PKA RII, and AKAP7 were found exclusively in the cytosolic fraction, while PKA RI was found predominantly in the membrane fraction (Figure 5A). Given that both PKA RI and RII were present in the cytosolic fraction, we sought to determine if either or both were associated with PDE3A. Immunoprecipitation of PDE3A demonstrated the association of PKA RII but not RI (Figure 3B), which was confirmed by reversed immunoprecipitations. We next performed a series of co-immunoprecipitation studies to determine the functional interactions of these proteins. In the first instance, we immunoprecipitated PDE3A and examined the presence of PKA regulatory isoforms, we again observed the association of PDE3A with PKA RII but not RI, AKAP7δ but not AKAP7γ, and PKAcat (Figure 5Bi). The immunoprecipitated complex was also shown to possess significant milrinone-sensitive PDE activity (Figure 5Bii). We next immunoprecipitated PKA RII and observed its association with PDE3A, PKAcat, and AKAP7δ (Figure 5Ci), along with milrinone-sensitive PDE activity (Figure 5Cii). Finally, immunoprecipitation of AKAP7 demonstrated that it was associated with PDE3A, PKAcat, and PKA RII (Figure 5Di) and possessed milrinone-sensitive PDE activity (Figure 5Dii). In experiments with PDE3A (UNT) antibody, we were unable to co-immunoprecipitate other proteins.

## 4. Discussion

PDE3A plays a key role in controlling intracellular cAMP concentrations in platelets, and therefore, its regulation is a critical link between platelet-driven haemostasis and thrombosis. Like most cells, platelets express multiple isoforms of key enzymes that control the cAMP response in cells. However, while there has been an explosion in our understanding of the spatiotemporal regulation of cAMP in nucleated cells [34,35], less is known about platelets given the limitations of using standard molecular biology approaches. We and others have provided the first evidence for the existence of cAMP signalling scaffolds and AKAPs being present in platelets [4,36,37], and here, we expand this work to identify the first PDE3A signalling complex in platelets. We demonstrate that (i) PDE3A isoforms are present in platelets but that the predominant forms are at 100 kDa protein in the cytosol, at basal; (ii) the phosphorylation and activation of cytosolic PDE3A in response to cAMP signalling is AKAP-dependent; (iii) AKAP7δ is expressed in platelets; and (iv) AKAP7δ scaffolds PKA RII to PDE3A to regulate its activity.

Platelet PDE3A is a target for multiple serine/threonine kinases that facilitate enhanced activity and protein–protein interactions [28]. PGI_2_ induces phosphorylation and activation of PDE3A through PKA, while agonists such as thrombin also phosphorylate and activate PDE3A through PKC and potentially Akt (PKB) [5]. However, within the constraints of our current knowledge, this dual context-dependent system for controlling PDE3A activity in platelets is difficult to reconcile, since both PKA and PKC activate PDE3A by targeting the same phosphorylation sites. We hypothesised that this could be explained by PKA and PKC targeting distinct and differentially localised PDE3A isoforms, a concept already highlighted in cardiomyocytes [21,30]. We generated three antibodies to establish this possibility in platelets. A PDE3A(NT) antibody that recognises the N-terminus (2–17aa) did not precipitate any protein, suggesting a full length PDE3A may not be expressed in platelets. Our biochemical and mass spectrometry data indicated the presence of other PDE3A isoforms, recognised by antibodies raised against C-terminus (1095–1110aa; PDE3A(CT)) and unique N-terminus regions (145–199aa; PDE3A(UNT)). The PDE3A(UNT) specific antibody precipitated a protein from platelets that was confirmed by both mass spectrometry and immunoblotting to be PDE3A. This protein had milrinone-sensitive activity but represented only 0.33 ± 0.03% of total platelet PDE3A activity. In contrast, immunoprecipitation with PDE3A(CT) was associated with robust basal PDE activity and was exclusively found in the soluble fraction. These data suggest that the PDE3A isoforms in platelets are distinct from other PDE3A expressing cells, for example, cardiomyocytes which express PDE3A1 (membrane), PDE3A2 (cytosolic), and PDE3A3 isoforms (both cytosolic and membrane) [26,38], while platelets only possess cytosolic PDE3A activity under basal conditions. In cardiomyocytes, PDE3A1 is present in the membrane fraction due to its unique N-terminal sequence [7,26,38]. However, we were unable to detect significant PDE3A1 activity or PDE3A protein in the particulate fraction. Furthermore, the PDE3A1 in platelets is found at around 100 kDa even though it contains the unique N-terminus as in cardiomyocytes, but the molecular weight of cardiomyocytes’ PDE3A1 is 118 kDa, suggesting post-translational modifications in megakaryocytes. Given our studies work with terminally differentiated cells, we were unable to use standard molecular biology approaches to alter the expression of PDE3A1; therefore, we focused our efforts on the much larger fraction of PDE3A isoforms and their regulation by PKA.

PGI_2_, the key regulator of platelets *in vivo*, induced the phosphorylation and activation of PDE3A in a cAMP- and PKA-dependent manner [5]. Critically, we observed that both phosphorylation and activation of PDE3A activity were sensitive to cell permeable AKAP-uncoupling peptides, suggesting that PDE3A was part of a complex with PKA. The use of two distinct peptide disrupters, HT31 and the PKA RI-specific disruptor RIAD, allowed us to determine that the phosphorylation of PDE3A in response to PGI_2_ lies downstream of a PKA RII subtype. Previously, we found that PKA RII is primarily located in the cytosol of platelets, suggesting it resides in the same compartment as PDE3A [4]. To confirm this, we fractionated platelets and confirmed that PKA RI was present in both fractions, while PKA RII was exclusively cytosolic. Having found data to suggest that PDE3A was phosphorylated by PKA RII in an AKAP-dependent manner, we sought to identify the AKAP responsible. We observed the presence of AKAP7, AKAP9, AKAP12, AKAP13, and moesin in human platelets. Of these, AKAP7 seemed the ideal candidate given that it has been shown to interact with PDE3A in cardiomyocytes [30]. After we confirmed the presence of AKAP7δ in both human and murine platelets by immunoblotting, we used a cAMP enrichment approach from platelet lysates to isolate cAMP binding protein complexes. Immunoblotting of proteins enriched by 8-AHA-cAMP-agarose beads demonstrated the presence of AKAP7. The confirmation that AKAP7 was acting as an AKAP was provided by two further observations. Firstly, immunoprecipitated AKAP7 possessed PKA catalytic activity, and secondly, AKAP7 and PKA RII were closely located in whole platelets in proximity ligation assay. Together, and consistent with other cell types [30,32], these data suggest that AKAP7 can act as an AKAP for PKA RII in platelets. In biochemical studies, the potential components of the PDE3A signalling complex were found to be in the cytosolic compartment of platelets. Co-immunoprecipitation of these components from the cytosolic fractions of platelets demonstrated that when PDE3A, PKA RII, and AKAP7 were precipitated from platelets, each of the other proteins were pulled down and, critically, all complexes contained milrinone-sensitive PDE activity.

The continual exposure of platelets to PGI_2_ in circulation leads to the stimulation of cAMP signalling, and the phosphorylation of a plethora of downstream effectors is required to ensure that platelets are tightly regulated. The targeting and activation of PDE3A catalytic activity in response to PGI_2_ likely forms a regulatory negative feedback loop which sustains cAMP levels at a threshold necessary to inhibit platelets whilst preserving their ability to become activated under thrombotic stimuli. To achieve this, the activity of PDE3A needs to be localised to ensure tight pools of cAMP. Translating this from nucleated cells like cardiomyocytes to platelets is difficult given that a compartment in a cardiomyocyte, for example, is similar in size to a platelet. Nevertheless, the current study sets an important precedent by identifying that PDE3A exists in a complex linked to AKAP7, and given the presence of multiple AKAPs, suggests the intriguing possibility that PDE3A may exist in multiple discreet complexes required to regulate distinct platelet functions. We have found that overactivity of PDE3A in platelets may be responsible for platelet hyperactivity linked to atherogenic lipid stress, and therefore, represents a potential antithrombotic target [5]. The use of PDE3A inhibitors such as Cilostazol is not new since they have been used in the clinical setting to control excess platelet activity. However, this and other inhibitors do not have sufficient specificity to target individual isoforms and lead to significant side effects [15]. A more nuanced strategy for selective targeting of PDE3A activities could be to displace PDE3A from specific signalling complexes, preventing recruitment and retainment in the signalling node.

## 5. Conclusions

Our data reveal the first evidence for spatiotemporal regulation of cAMP signalling in blood platelets by the presence of a PDE3A/PKA RII/AKAP7 signalling complex. This new information may allow the development of new strategies to target specific PDE3A activity in platelets to control thrombosis.

## Data Availability

The original contributions presented in this study are included in the article/Supplementary Material, further inquiries can be directed to the corresponding author.

## Supplementary Materials

The following supporting information can be downloaded at https://www.mdpi.com/article/10.3390/cells13131104/s1, Figure S1. PDE3A inhibitor milrinone potentiates PGI_2_ stimulated platelet inhibition; Figure S2. Mass spectrometry analysis of immunoprecipitated PDE3A protein bands; Figure S3. Densitometric quantification of immunoblots from Figure 3Ai; Figure S4. Functional effect of AKAP7–PKA RII interaction on PGI_2_ stimulated platelet inhibition; Figure S5. Expressions of AKAP9, AKAP12, AKAP13, and moesin in human platelets; Table S1: Mass spectrometry analysis of immunoprecipitated PDE3A protein bands.
