# Supplementary material for: Protein Kinase A Regulates Platelet Phosphodiesterase 3A through an A-Kinase Anchoring Protein Dependent Manner
Source: Cells. 2024 Jun 26;13(13):1104. doi: 10.3390/cells13131104 (PMC11240354; doi:10.3390/cells13131104)
Supplement: Supplementary file 1 [file cells-13-01104-s001.zip › Cells-PDE3A-AKAP7 Supplementary Figures.pdf]

Article

Protein Kinase A regulates platelet phosphodiesterase 3A through an A-kinase anchoring protein dependent manner

Supplementary Materials

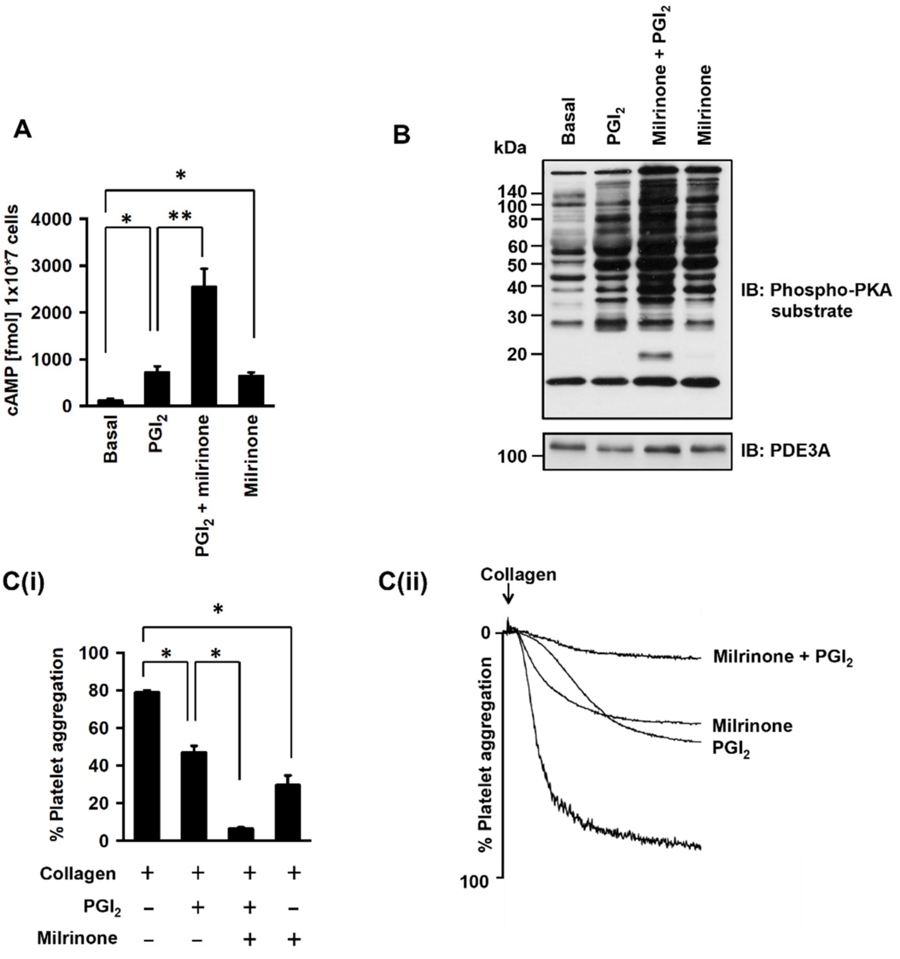

Figure S1. PDE3A inhibitor milrinone potentiates PGI_2 stimulated platelet inhibition.

A Washed platelets (2x10^8 platelets/ml) were treated with and without PGI_2 (10nM, 2 minutes) and milrinone (10μM, 20 minutes). Intracellular cAMP concentrations were measured (n=3) *P<0.01. **B** Sample treatment as in A, and PKA substrate phosphorylation events were assessed by immunoblotting. Blots were stripped and reprobed for PDE3A as a protein loading control. Representative immunoblot of 3 independent experiments. **C** Washed platelets (2.5x10^8 platelets/ml) were stimulated with collagen (5μg/ml) in the presence and absence of PGI_2 (10nM, 2 minutes) and milrinone (10μM, 20 minutes). Aggregation traces were recorded for 5 minutes. **C(i)** Data are expressed as % platelet aggregation, and **C(ii)** representative aggregation traces of 3 independent experiments *P<0.01.

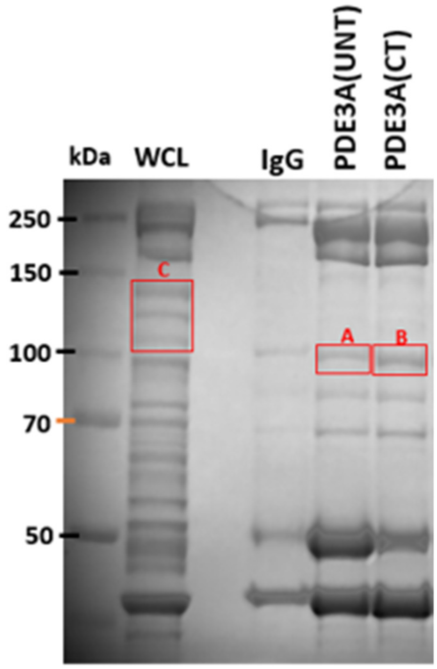

Figure S2. Mass spectrometry analysis of immunoprecipitated PDE3A protein bands.

Washed platelets (8x10^8 platelets/ml) were lysed with IP lysis buffer, PDE3A proteins (500µg) were immunoprecipitated with PDE3A(CT) and PDE3A(UNT) antibodies. Immunoprecipitated and whole cell lysate samples were run on SDS/PAGE prior to Coomassie staining of a gel. Boxes A, B, and C were drawn to show where the gel was excised and analysed via mass spectrometry.

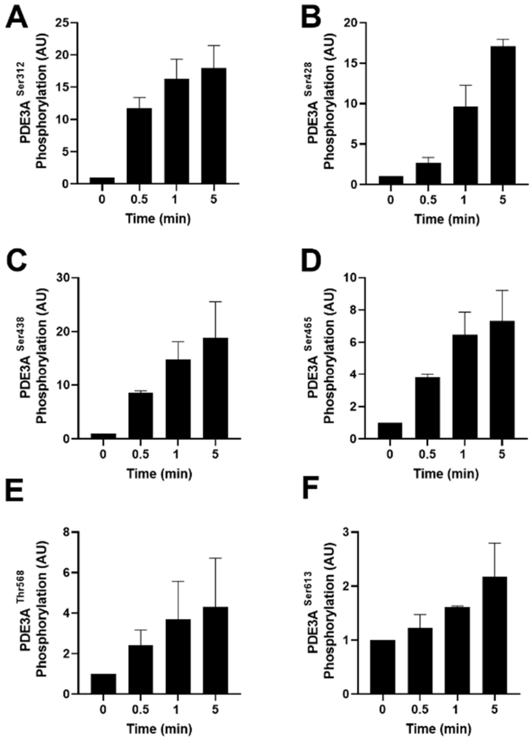

Figure S3. Densitometric quantification of immunoblots from Figure 3Ai.

Densitometric analysis PDE3A phosphorylation at sites ser312 (**A**), ser428 (**B**), ser438 (**C**), ser465 (**D**), thr568 (**E**) and ser613 (**F**) and expressed as arbitrary unit (AU).

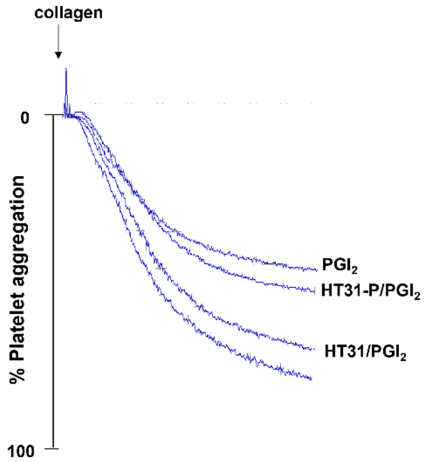

Figure S4. Functional effect of AKAP7 – PKA RII interaction on PGI_2 stimulated platelet inhibition.

Washed platelets (2.5x10^8 platelets/ml) were stimulated with collagen (5µg/ml) in the presence and absence of PGI_2 (10nM, 2 minutes), pre-treated with non-specific AKAP disruptor peptide HT31 (2µM, 20 minutes) or control peptide HT31-P (2µM, 20 minutes). Aggregation traces were recorded for 5 minutes. Data are expressed as % platelet aggregation.

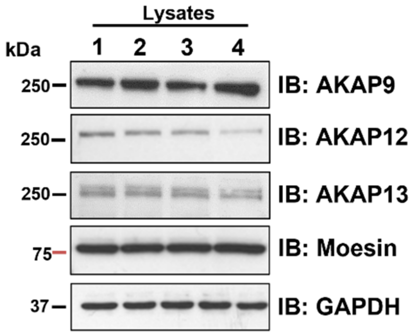

Figure S5. Expressions of AKAP9, AKAP12, AKAP13 and moesin in human platelets.

Washed platelet lysates (5x10^8 platelets/ml) were separated by SDS-PAGE and immunoblotted for AKAP9, AKAP12, AKAP13 and moesin. GAPDH was used as loading control.

(A) PDE3A(UNT) (100kDa)	(B) PDE3A(CT) (100kDa)	(C) WCL (130kDa, non specific band)
Echinoderm microtubule-associated protein-like 3	**cGMP-inhibited 3',5'-cyclic phosphodiesterase A**	Vinculin
Rho GTPase-activating protein 6	Integrin alpha-Iib	Thrombospondin-1
Nexilin	LIM domain and actin-binding protein 1	Talin-1
Oligophrenin-1	Rho GTPase-activating protein 6	Filamin-A
Villin-1	Villin-1	Protein unc-13 homolog D
LIM domain and actin-binding protein 1	Sarcoplasmic/endoplasmic reticulum calcium ATPase 2	Integrin alpha-6
Sarcoplasmic/endoplasmic reticulum calcium ATPase 2	Oligophrenin-1	Integrin alpha-Iib
Zinc finger CCCH-type antiviral protein 1	Actin, alpha cardiac muscle 1	WD repeat-containing protein 44
Protein unc-45 homolog A	Protein unc-45 homolog A	ATP-citrate synthase
Myosin-14	Myosin-14	Myosin-9
Protein MTSS 1	Zinc finger CCCH-type antiviral protein 1	Extended synaptotagmin-1
Gelsolin	Gelsolin	Kinesin-1 heavy chain
Integrin alpha-Iib	FH1/FH2 domain-containing protein 1	Platelet endothelial cell adhesion molecule
Protein SLFN14	Transitional endoplasmic reticulum ATPase	Complement C3
KN motif and ankyrin repeat domain-containing protein 2	cGMP-specific 3',5'-cyclic phosphodiesterase	Integrin beta-1
FH1/FH2 domain-containing protein 1	Nexilin	Protein phosphatase 1 regulatory subunit 12A
Complement C4-A	Immunoglobulin heavy constant gamma 4	Arf-GAP with SH3 domain, ANK repeat and PH domain-containing protein 2
Protein flightless-1 homolog	Protein MTSS 1	Leucine-rich repeat flightless-interacting protein 1
Tropomyosin alpha-4 chain	Vinculin	Sarcoplasmic/endoplasmic reticulum calcium ATPase 3
WD repeat-containing protein 1	Protein SLFN14	Sarcoplasmic/endoplasmic reticulum calcium ATPase 2
Protein phosphatase 1 regulatory subunit 12A	Coatomer subunit beta'	Latent-transforming growth factor beta-binding protein 1
Thrombospondin-2	Protein flightless-1 homolog	Nck-associated protein 1
cGMP-specific 3',5'-cyclic phosphodiesterase	Unconventional myosin-Ic	Platelet glycoprotein Ib alpha chain
Taperin	FYVE, RhoGEF and PH domain-containing protein	CAP-Gly domain-containing linker protein 2
Epidermal growth factor receptor substrate 15-like 1	Coatomer subunit gamma-1	Ras GTPase-activating protein 1
**cGMP-inhibited 3',5'-cyclic phosphodiesterase A**	Complement C4-A	Keratin, type I cytoskeletal 10
Immunoglobulin heavy variable 3/OR16-9 (non-functional) (Fragment)	Echinoderm microtubule-associated protein-like 3	P-selectin
Transitional endoplasmic reticulum ATPase	Protein phosphatase 1 regulatory subunit 12A	DNA damage-binding protein 1
NLR family member X1	Hexokinase-1	Disheveled-associated activator of morphogenesis 1
Unconventional myosin-Ic	AP-1 complex subunit beta-1	Epidermal growth factor receptor substrate 15

All the listed proteins were detected/identified with a confidence threshold greater than 95% ($p<0.05$). Only the top 30 proteins are listed, the rest of the proteins can be found in supplementary excel spreadsheet.

Table S1. Mass spectrometry analysis of immunoprecipitated PDE3A protein bands.

Top 30 proteins found in respective excised gel sections (A, B and C) were listed. The rest of the proteins can be found in supplementary excel spreadsheet.
